# Characterization of Injuries Suffered by Mounted and Non-Mounted Police Officers

**DOI:** 10.3390/ijerph20021144

**Published:** 2023-01-09

**Authors:** Robin Orr, Elisa F. D. Canetti, Rodney Pope, Robert G. Lockie, J. Jay Dawes, Ben Schram

**Affiliations:** 1 Tactical Research Unit, Bond University, Robina, QLD 4226, Australia; 2 Faculty of Health Sciences and Medicine, Bond University, Robina, QLD 4226, Australia; 3 School of Allied Health, Exercise and Sports Sciences, Charles Sturt University, Albury, NSW 2640, Australia; 4 Department of Kinesiology, California State University, Fullerton, CA 92831, USA; 5 School of Kinesiology, Applied Health and Recreation, Oklahoma State University, Stillwater, OK 74078, USA; 6 OSU Tactical Fitness and Nutrition Lab, Oklahoma State University, Stillwater, OK 74078, USA

**Keywords:** law enforcement, horses, back pain, occupation, policing, equestrian

## Abstract

Mounted police officers are subject to unique occupational tasks which may lead to unique injuries. This study’s aim was to describe policing injuries suffered by mounted police officers contextualized through comparison to non-mounted officers. Injury data from 01 July 2014 to 30 June 2020 were provided from a state policing agency’s incident reporting database. The data reported the numbers and rates of injuries and classified the injuries by gender, cause, mechanism, nature, and hours worked. Of the 35,406 reported injuries, 35,255 (99.6%) injuries were reported by non-mounted police officers. An annual incidence rate of 338–364 and 626–952 injuries per 1000 personnel were reported in non-mounted and mounted police, respectively. For mounted police, the leading causes of injuries were slips, trips, and falls (23.8%), followed by repetitive tasks and movements (9.9%). Physical assault was the leading cause of injury for non-mounted police officers (21.3%), followed by slips, trips, and falls (16.0%). In mounted police, falls from heights (15.9%) and repetitive tasks and movements (10.6%) comprised the most frequently specified mechanisms of injury, as compared to physical assault (21.0%) and physical exercise (5.2%) in non-mounted police. The most common activities being performed at the time of injury for mounted police were animal handling (64.9%) as opposed to arresting an offender (31.2%) for non-mounted police. Sprains and strains and bruises and swelling were the leading natures of injuries among both mounted (44.4% and 29.1%, respectively) and non-mounted (36.6% and 21.2%, respectively) officers. The leading body sites of injury in mounted officers were the lower back (13.9%) and neck and shoulders (7.3% each), and for non-mounted police, the knee (13.9%), lower back (10.0%), and hand (8.2%) were the most common. Mounted police officers sustained injuries through different activities, causes, and mechanisms and to different body sites at 2–3 times higher incidence rates. Mounted police officers warrant specifically tailored injury mitigation and return-to-work strategies.

## 1. Introduction

Police officers are expected to perform a variety of occupational tasks. These tasks range from checking an individual’s identity to chasing and apprehending a fleeing suspect [1]. This variety means that a police officer could experience situations that greatly vary in physical demands, ranging from those that are predominantly sedentary (e.g., desk work and communications) to those that are high intensity in nature (e.g., grappling with a suspect) [1]. To add to the potential physical demand, police officers often have to perform these tasks while carrying extra weight on their persons (up to 10 kg) comprising vital gear and safety equipment [2]. Additionally, these physically demanding tasks often occur with little to no warning, limiting the ability of an officer to prepare themselves mentally and physically [3]. This combination of factors is a key reason why police officers are at an increased risk of injury [4].

A critical review by Lyons et al. [4] found that police officers suffered injuries at a rate ranging from 240 to 2500 per 1000 personnel per year, a higher injury rate than some other physically demanding occupations, such as mining, which has shown a rate of 164 to 638 injuries per 1000 people per year [5]. The review found that injuries commonly affected the upper limb and back and were mainly musculoskeletal in nature [4]. These injuries have multiple downstream effects on both organizations and individuals. At the organizational level, injuries increase healthcare costs (to provide sufficient care for the officers) [6] and increase workforce strain [7]. If an officer is unable to work, other officers would need to work more hours, adding to their workload and potentially increasing their risk of injury. Individually, injuries could affect career longevity by increasing the risk of future injury [8] and impairing performance upon return to full-time duty [9]. While sports teams may offer some control over the rehabilitation of players, police officers work in a naturally unpredictable environment and perform a wide variety of different tasks [1,10]. Given their role in protecting the public, decreases in officers’ performance could lead to severe injury or even death of themselves, their colleagues, or members of the community they serve [11,12]. Due to the significant impacts injuries have on both organizations and personnel, it is vital that injury mitigation strategies are developed and implemented. 

Injury mitigation strategies should be specifically created for their target population [13]. Although the review by Lyons et al. [4] reported a general injury profile for police officers, injuries may vary across roles and responsibilities. For example, specialist police are more likely to experience back injuries [6], and individual studies of various police forces have found the lower limb to be the most common site of injury [14]. Given the wide range of roles that exist in a police department, from general police to water police and specialist tactical groups [1,15,16], it is unlikely that all facets of a department would experience the same injuries in the same fashion. As such, specific strategies need to be designed to address the specific sources of injury risk within each group. One group that has received little scientific analysis is the mounted police. 

Mounted police are unique in their job demands as, in addition to their general duties, they must also look after and work with horses [17,18]. Apart from the anticipated unique duties like grooming and caring for their mounts, saddlery, and cleaning stables, operational and special duties tasks may see mounted officers patrolling parks and streets on horseback, working as mounted crowd and traffic control, and even working in livestock mustering [17,18]. These officers and their mounts may also conduct ceremonial and public relations activities, like parades, escorts, vaulting exhibitions, and official excursions [17,18]. Due to the uniqueness of this position, it cannot be reasonably assumed that mounted police would experience similar injuries in the same manner as general police. Therefore, the aim of this study was to describe policing injuries suffered by mounted police officers. To contextualize this data, a comparison of injuries among non-mounted officers was also conducted using the data from non-mounted police of the same organization. It was hypothesized that mounted police officers would report different injuries to non-mounted officers and, as such, require different injury mitigation strategies.

## 2. Materials and Methods

The study was designed as a population-based, longitudinal study using data previously collected prospectively by an Australian state police force. Injury incident data relating to preceding years were provided in a spreadsheet from the police force’s incident reporting database. The provided data set contained records related to the frequencies of injury incidents that occurred in the mounted and non-mounted elements of the police force within each financial year between 01 July 2014 (the financial year of 2014/15) and 30 June 2020 (the financial year of 2019/20). These frequencies were also broken down by gender, reported activity performed at the time of injury, injury cause, injury mechanism, injury nature, affected body site, and hours worked prior to the injury. In relation to this study, the “activity” was the task being undertaken at the time of injury, the “cause” was considered the action that best described the circumstances that resulted in the injury, while the “mechanism” described how the “cause” was delivered. As an example, for an officer bitten by a horse when leading it from the stables, the activity would be listed as “animal handling”, the cause would be listed as “animal bite”, and the mechanism was “horse”. For an officer who tripped walking up the steps to their office, the activity could be “general duties”, while the cause would be “slips, trips, and falls”, and the mechanism would be “steps/staircase”. The nature of an injury describes the type of injury that occurred (e.g., a ligament sprain or muscle strain), while the body location describes where on the body the type of injury occurred. In addition, the data indicating the annual injury incidence rates in both the mounted and non-mounted elements of the police force were provided as part of the data set. Ethics approval for the study was provided by the Bond University Human Research Ethics Committee (BS02126).

The participants from whom the data had been derived were current active officers within an Australian state police force during the timeframe in which the data were prospectively recorded by the New South Wales police force in the database from which they were extracted. Given the retrospective nature of the data, information regarding the height, weight, or age of the injured officers was not provided, and data regarding the proportions of the underlying mounted and non-mounted police populations that were of each gender or associated with other demographic characteristics were also unavailable. This lack of demographic information is common in tactical populations, often due to security concerns [19,20]. 

The data were analyzed descriptively to derive, for each of the mounted and non-mounted elements of the police force, the annual injury incidence rates reported per 1000 personnel and the proportions of injuries that were associated with each gender and with specific reported activities performed at the time of injury, injury causes, injury mechanisms, injury nature, affected body sites, and hours worked prior to the injury. The calculation of the annual injury incidence rates per 1000 personnel as the denominator allowed for a ready comparison to the research conducted in other law enforcement populations [4]. Calculations of confidence intervals around the rates, proportions, and significance testing of the differences between the mounted and non-mounted elements of the police force were not performed because this was not appropriate; specifically, the data used in the study were drawn from the entire population of mounted and non-mounted police officers within the state’s police force in the time periods of interest rather than being drawn from samples derived from these populations, and so there was no uncertainty in the population estimates of the rates, proportions, and differences derived from the analysis (which there would have been if the data were drawn from samples derived from the two police populations). This meant that neither calculation of the precision of the population estimates nor the inferential statistics (to derive inferences from a sample for the underlying population) were appropriate or required. 

## 3. Results

A total of 35,406 injuries were reported. Of these, 151 (0.4%) were reported by mounted police officers, who comprised a single unit within the state’s police force, and 35,255 (99.6%) injuries were reported by non-mounted police officers. The annual injury incidence rates for the mounted officers ranged from 626 to 952 injuries per 1000 personnel, and these rates were substantially higher than the annual injury incidence rates for the non-mounted officers, which ranged from 338 to 364 injuries per 1000 personnel (Table 1).

Male officers sustained 76% of non-mounted officer injuries, while 82% of the mounted police unit injuries were to female officers. However, information regarding the proportions of the underlying population comprising each element by each gender was not available. Thus, it was not possible to ascertain the injury incidence rates for each gender within each of the mounted and non-mounted elements or determine whether and to what extent these differed. 

The most commonly specified activities performed at the time of injury for the mounted officers were animal handling (64.9%) and police training (6.0%). For non-mounted officers, the most commonly specified activities were arresting an offender (31.2%) and general duties (9.9%) (Table 2).

In the mounted unit, the most commonly specified cause of injuries was slips, trips, and falls (23.8%). Repetitive tasks and movements and being struck by an object were also prevalent causes of injury in the mounted police (9.9% and 7.3%, respectively). Physical assault was the specified leading cause of injury for the non-mounted police officers (21.3%), followed by slips, trips, and falls (16.0%) and physical exercise (5.8%) (Table 3).

In mounted police, falls from heights (15.9%), repetitive tasks (10.6%), and horses (9.3%) were the most commonly specified injury mechanisms. In the non-mounted police officers, the most prevalent specified mechanisms of injuries were physical assault (21.0%), physical exercise (5.2%), and uneven surfaces (5.2%) (Table 4).

Sprains and strains and “bruises and swelling” were the leading natures of injuries for both mounted (44.4% and 29.1%, respectively) and non-mounted (36.6% and 21.2%, respectively) personnel (Table 5).

The mounted officers mostly reported suffering injuries to their lower back (13.9%), neck (7.3%), and shoulders (7.3%), while the non-mounted officers reported suffering injuries to their knees (13.9%), lower back (10.0%), and hands (8.2%) (Table 6).

Most injuries occurred between 4 and 12.5 h after a shift commenced (67.3%) for the non-mounted police, with more than half of those (34.5%) occurring after 8 h on the job (Table 6). In the mounted police, most injuries occurred within eight hours of the shift commencing (72.2%), with 41.1% occurring within the first four hours of the shift (Table 7).

## 4. Discussion

The aim of this study was to describe the policing injuries suffered by mounted police officers. Notably, mounted police officers suffered injuries at approximately two to three times higher rates than non-mounted police, with the incidence rates suggesting that the majority of mounted officers suffered injuries each year. Similar to the non-mounted police officers, the mounted officers were found to experience sprains, strains, bruises, and swelling as their most common nature of injury. However, the profiles of the activities performed at the time of injury occurrence, the reported causes and mechanisms of injury, and the body sites of injury were notably different in the mounted police officers when compared to the non-mounted police officers. 

For the mounted police officers, the incidence rate for the injuries reported in this study ranged from 626 to 952 injuries per 1000 personnel per year, a rate notably higher than that for non-mounted officers, ranging from 338 to 364 injuries per 1000 personnel per year. A review of the injuries in law enforcement populations by Lyons et al. [4] found that the reported incidence of injuries among law enforcement personnel varied from 240 [21] to 2500 [22] injuries per 1000 person-years. While both the mounted and non-mounted incidence rates observed in this study fell within this range, variations in the reported injury incidence rates between the studies included in the review could, in many instances, be attributed to research design factors, such as the included injury types, nature of the studies compared, sources of data, data collection procedures (e.g., self-reported, workplace databases, etc.), and injury definitions employed [4]. Regardless, given that in this study, the data for both mounted and non-mounted police originated from the same organization using the same data collection protocols, the nearly two to three times higher incidence rate for the injuries in mounted police as opposed to their non-mounted counterparts likely represents a true difference and is of concern.

Although making up a higher percentage of injuries in mounted police officers when compared to non-mounted officers (44.4% versus 36.6%), the leading nature of injuries in both elements was musculoskeletal sprains and strains. This finding is supported by the wider literature detailing injuries in law enforcement [4]. Following sprains and strains, both mounted and non-mounted officers next most commonly reported suffering bruises and swelling. Again, the proportion of all injuries these represented was higher in mounted than non-mounted officers (29.1% versus 21.2%).

When considering the activity reportedly being undertaken at the time of injury, animal handling was the leading activity associated with injuries suffered by mounted officers, accounting for 64.9% of injuries, followed by police training (6.0%). The specified leading causes of injury were a slip, trip, or fall (23.8%) or repetitive tasks and movements (9.9%). The specified leading mechanism of injury was a fall from heights (15.9%), followed by repetitive tasks and movements (10.6%). There appears to be a good alignment between the reported activities, causes, and mechanisms involved in the injury occurrences. For example, mounted police training would likely involve interactions with their equine mounts and slips, trips, and falls, and repetitive tasks and movements could be associated with both horse handling and mounted police training. However, it should also be noted that for mounted police, 33% of injury causes and 25% of injury mechanisms were reported as “other or unspecified”; this may indicate that many actual causes and mechanisms were difficult to code using existing categories in the database and future research would benefit from open-ended questions allowing free text responses that enabled the accurate reporting of the causes and mechanisms in the mounted police. Regardless, the activities most commonly undertaken at the time of injury in mounted police, as well as the causes and mechanisms of injury, were different from those most commonly reported in non-mounted police; the most common activity in the latter was arresting an offender (31.2%), with physical assault being both the leading cause (21.3%) and mechanism (21.0%) of injury. Dealing with an offender is typically a leading mechanism of injury reported by police in the wider literature [4,23], and as such, it is not unexpected for the non-mounted officers in this study. Finally, while slips, trips, and falls were also the second most common cause of injury reported in non-mounted officers, they represented only 16.0% of the reported injuries, as opposed to the 23.8% of injuries for which they were the reported cause in the mounted police.

The body sites of injuries reported in non-mounted officers were similar to those previously reported in the literature [4]. Previous research in New Zealand [24] and Canadian [23] police and in a literature review reporting on several agencies [4] found the upper extremity was the leading body site of injury in law enforcement. In this study, four of the top six most common body sites of injuries, equating to over a quarter of injuries (26.7%), were to the upper extremity, with the knee (13.9%) and lower back (10.0%) also commonly reported as injured in the non-mounted police. A study reporting on lower limb injuries, specifically in an Australian state police force, also identified the knee as the leading body site of lower limb injury [25], consistent with the findings of this study. 

In contrast to the non-mounted police and findings from the non-mounted police forces reported in the broader literature, the mounted police officers’ leading body sites of injury were different, with the lower back (13.9%) followed by the neck (7.3%) reported as the leading body sites of injury. Thus, the top two sites of bodily injury for mounted officers (together representing 21.2% of the injuries) were the torso, which includes the spinal system. Of note, a study by Hua et al. [26] investigating the injuries suffered by police officers seeking in-house physiotherapy treatments noted that the leading body site of injury was the lower back, accounting for 40.5% of the injuries and 40.2% of all treatment sessions. Also, unlike the non-mounted officers, the leading site of lower extremity injury in the mounted officers was the ankle (mounted = 6.6%; non-mounted = 3.9%) rather than the knee (mounted = 4.0%; non-mounted officers = 13.9%). Thus, the leading body sites of injuries reported by the mounted police officers differed from those reported by the non-mounted police in this study and were typically reported by non-mounted police considered in the broader literature. These differences may arise due to differences in tasks performed and risks faced by the mounted police compared to non-mounted police, and further research is warranted to explore this possibility further.

Finally, the mounted police officers appeared more likely to suffer injuries during the first four hours of a twelve-hour shift (41.1%) than in the last four hours (27.2%). This differed from the non-mounted officers, whose injuries were fairly evenly spread across the shift. Potential reasons for this difference are unclear, and further investigation is warranted.

While this is the first known investigation to compare the injuries between mounted and non-mounted police officers, several limitations should be acknowledged. First, the injury data were limited to one police agency in one Australian state. Consequently, the types of injuries sustained may have been influenced by the frequency and types of incidents officers were required to respond to in this region. Thus, the injury types and frequencies may vary among other organizations based on population size, policing techniques, and local laws. Additionally, this analysis is based on those injuries reported. Injuries considered minor in severity or those not requiring medical care may not have been reported and instead may have been self-managed, as is common in tactical populations [27]. Finally, as the proportions of the underlying population comprising each element that was of each gender were not available, it was not possible to further explore the injury incidence rates for each gender nor compare these rates across the different law enforcement elements.

## 5. Conclusions

Mounted police officer injury profiles differed in a variety of ways from those of non-mounted police officers, and the injuries appeared to occur at a substantially higher rate in the mounted police. These results highlight a need to approach injury prevention and mitigation programs differently in mounted police units as opposed to the general duties of officers and denote potentially different injury rehabilitation and return-to-work requirements. For example, with the torso and spine (notably the lower back) being the leading body sites of injury in the mounted police and the nature of these injuries, most commonly musculoskeletal in nature, means of reducing the musculoskeletal load to the lower back, deserve investigation. Likewise, the means of optimizing the physical resilience of the lower back (e.g., strengthening of the trunk) may be of benefit. Finally, to better inform research specific to this population, a review of the police injury database and data entry procedures should be undertaken with a focus on reducing or supplementing with open-ended questions the use of the “other” category for recording both the causes and mechanisms of injury.

## Figures and Tables

**Table 1:** Annual injury incidence rates by police force element and financial year.

Financial Year	Mounted Officers		Non-Mounted Officers	
	Number of Incidents	Incidence Rate (per 1000 Personnel)	Number of Incidents	Incidence Rate (per 1000 Personnel)
2014–15	30	952	5705	344
2015–16	26	761	5622	338
2016–17	27	770	5931	356
2017–18	22	626	5821	348
2018–19	24	701	6151	364
2019–20	22	638	6025	351
Mean 2014–20	25.17 ± 3.13	741.33 ± 119.33	5875.83 ± 198.81	350.17 ± 9.13

**Table 2:** Injuries by reported activity at the time of the incident.

Mounted Officers			Non-Mounted Officers		
Injuries	Percentage	Activity at the Time of the Incident	Injuries	Percentage	Activity at the Time of the Incident
98	64.9%	Animal handling	10,988	31.2%	Arresting an offender
19	12.6%	Other or unspecified	3505	9.9%	Other or unspecified
9	6.0%	Police training	3492	9.9%	General duties
6	4.0%	Manual handling	2306	6.5%	Restraining an offender
5	3.3%	Walking, running	1715	4.9%	Walking, running
4	2.7%	Arresting an offender	1645	4.7%	Pursuit on foot
3	2.0%	Journey to or from work	1266	3.6%	Station office duties
2	1.3%	Crowd control	1044	3.0%	Police training
2	1.3%	Station office duties	1047	3.0%	Journey to or from work
1	0.7%	General duties	1016	2.9%	Mental health intervention
1	0.7%	Non-work-related incident	969	2.8%	Manual handling
1	0.7%	Weapons training	818	2.3%	Weapons training
			646	1.8%	Search persons/possess
			613	1.7%	Patrol in vehicle
			386	1.1%	Animal handling
			463	1.3%	Patrol on foot
			442	1.3%	Journey on duty
			302	0.9%	OSG training
			275	0.8%	Attending MVA
			250	0.7%	Crowd control
			221	0.6%	Special operations (OSG)
			207	0.6%	Search premises/vehicle
			204	0.6%	Search and rescue
			177	0.5%	Traffic control
			175	0.5%	Diving
			174	0.6%	Patrol on bicycle
			171	0.5%	Patrol on motorcycle
			145	0.4%	Pursuit in vehicle
			115	0.3%	Evacuation
			114	0.3%	Drug or alcohol-related
			114	0.3%	Technical support
			106	0.3%	Boat handling
			81	0.2%	Non-work-related incident
			26	0.1%	Putting on PPE
			19	0.1%	Working at heights
			5	0.01%	Pursuit by motorcycle
			4	0.01%	Bicycle urgent duty
			4	0.01%	Handling biochemical waste
			3	0.01%	Office duties (not station)
			2	0.01%	Parking patrol
151	100%		35,255	100%	

Key: MVA: motor vehicle accident; OSG: operations support group; PPE: personal protective equipment.

**Table 3:** Injuries by reported cause of the incident.

Mounted Officers			Non-Mounted Officers		
Number	Percentage	Cause of Incident	Number	Percentage	Cause of Incident
50	33.1%	Other or unspecified	7495	21.3%	Physical assault
36	23.8%	Slips, trips, and falls	5639	16.0%	Slips, trips, and falls
15	9.9%	Repetitive tasks and movements	5548	15.7%	Other or unspecified
11	7.3%	Struck by an object	2041	5.8%	Physical exercise
9	6.00%	Physical exercise	1933	5.5%	Manual handling
7	4.6%	Manual handling	1496	4.2%	Exposure to bodily fluids
6	4.00%	Animal bites	1312	3.7%	Motor vehicle collision
5	3.3%	Caught in or between objects	1322	3.8%	Repetitive tasks and movements
3	2.0%	Physical assault	1145	3.3%	Struck by an object
3	2.0%	Step on/striking an object	1079	3.1%	Step on/striking an object
2	1.3%	Rubbing and chafing	1039	3.0%	Entry/exit vehicle/premises
1	0.7%	Exposure to workplace violence	1019	2.9%	Exposure to workplace violence
1	0.7%	MVA	744	2.1%	Caught in or between object
1	0.7%	OC spray	562	1.6%	Animal bites
1	0.7%	Safety equipment	324	0.9%	OC spray
			301	0.9%	Human bites
			296	0.8%	Exposure to chemicals
			250	0.7%	Exposure to biological factors
			226	0.6%	Motorcycle collision
			211	0.6%	Bicycle accident
			197	0.6%	Insect bites and stings
			181	0.5%	Psychological factors
			125	0.4%	Needle-stick injury
			123	0.4%	Exposure to noise or vibrations
			114	0.3%	Exposure to gas or fumes
			107	0.3%	Other transport
			99	0.3%	Gun discharged
			90	0.3%	Rubbing and chafing
			89	0.3%	Exposure to high temperatures
			83	0.2%	Safety equipment
			22	0.1%	Electric shock
			21	0.1%	Explosion or implosion
			19	0.1%	PPE failure
			2	0.01%	Exposure to radiation
151	100%		35,255	100%	

Key: MVA: motor vehicle accident; OC spray: oleoresin capsicum spray; PPE: personal protective equipment.

**Table 4:** Injuries by reported mechanism.

Mounted Officers			Non-Mounted Officers		
Injuries	Percentage	Mechanism	Injuries	Percentage	Mechanism
38	25.2%	Other or unspecified	7385	21.0%	Physical assault
24	15.9%	Falls from a height	5329	15.1%	Other or unspecified
16	10.6%	Repetitive tasks and movements	1832	5.2%	Physical exercise
14	9.3%	Horses	1815	5.2%	Uneven surface
14	9.3%	Nature of work	1464	4.2%	Lifting/carrying/putting down an object
14	9.3%	Struck by an object	1350	3.8%	Stepping on/striking an object
5	3.3%	Caught in or between object	1211	3.4%	Body fluids and blood
4	2.7%	Manual handling	1203	3.4%	Repetitive tasks and movements
4	2.7%	Steps/stairway	1166	3.3%	Car
4	2.7%	Uneven surface	1091	3.01%	Struck by an object
2	1.3%	Loose surface	961	2.7%	Workplace violence
2	1.3%	Physical assault	902	2.6%	Steps/stairway
2	1.3%	Rubbing or chafing	871	2.5%	Nature of work
2	1.3%	Stepping on/striking an object	711	2.0%	Uniform (belt, vest, etc.)
1	0.7%	Car	676	1.9%	Body fluids and saliva
1	0.7%	OC spray	658	1.9%	Sharp object (other than a needle)
1	0.7%	Physical exercise	478	1.4%	Wet/oily/icy surface
1	0.7%	Safety (inc. P.P.E.)	475	1.4%	Falls from a height
1	0.7%	Uniform (belt, vest etc.)	463	1.3%	Dogs
1	0.7%	Unknown	449	1.3%	Entry/exit vehicle
			408	1.2%	Caught in or between object
			404	1.2%	Unknown
			375	1.1%	Loose surface
			340	1.0%	OC spray effects
			263	0.8%	Human bites
			242	0.7%	Entry/exit premises
			238	0.7%	Chemicals
			218	0.6%	Bicycle accident
			210	0.6%	Other biological factors
			200	0.6%	Fire
			200	0.6%	Hole in the ground
			179	0.5%	Road bike
			178	0.5%	Single attendant at a specific traumatic event
			167	0.5%	Insects
			163	0.5%	Body fluids
			110	0.3%	Gas fumes
			102	0.3%	Needle stick injury
			93	0.3%	Rubbing/chafing
			93	0.3%	Gun discharged
			93	0.3%	Noise/vibration
			77	0.2%	Extreme temperatures
			64	0.2%	Safety (including PPE)
			52	0.2%	Organizational
			33	0.1%	Trail bike
			26	0.1%	Truck
			25	0.1%	Spiders
			24	0.1%	Multiple attendances at trauma sites
			23	0.1%	Cats
			22	0.1%	Electric shock
			15	0.04%	Org-internal investigation
			15	0.04%	Org-performance management
			15	0.04%	Trapped in a confined space
			14	0.04%	Explosion or implosion
			13	0.04%	Boat
			13	0.04%	Electronic discharge weapon (taser)
			10	0.03%	Organization-staffing workload
			8	0.02%	Bus
			6	0.02%	Horses
			6	0.02%	Variation in pressure (other than sound)
			5	0.01%	Snow vehicle
			4	0.01%	Marine animals
			4	0.01%	Organizational-discipline/dismissal
			3	0.01%	Caving/landslide
			3	0.01%	Snakes
			3	0.01%	Train
			2	0.01%	Organizational-transfer/redundancy
			2	0.01%	Radiation
			1	0.00%	Improvised explosive device
			1	0.00%	Slips, trips, and falls
151	100%		35,525	100%	

Key: PPE: personal protective equipment; OC spray: oleoresin capsicum spray.

**Table 5:** Injuries by reported nature.

Mounted Officers			Non-Mounted Officers		
Injuries	Percentage	Main Nature of Injury	Injuries	Percentage	Main Nature of Injury
67	44.4%	Sprains and strains	12,887	36.6%	Sprains and strains
44	29.1%	Bruises and swelling	7472	21.2%	Bruises and swelling
15	9.9%	Other or unspecified	4107	11.7%	Abrasions and superficial injuries
9	6.0%	Abrasions and superficial injuries	3757	10.7%	Other or unspecified
4	2.7%	Lacerations or open wounds	2469	7.0%	Laceration
3	2.0%	Internal injury	1079	3.1%	Internal injury
2	1.3%	Concussion and cranial injuries	817	2.3%	Fracture or dislocation
2	1.3%	Fracture or dislocation	795	2.3%	Infectious diseases
2	1.3%	OOS	428	1.2%	Foreign bodies
1	0.7%	Burns and scalds	209	0.6%	Respiratory disease
1	0.7%	Eye disorders	196	0.6%	Burns and scalds
1	0.7%	Multiple injuries	179	0.5%	Multiple injuries
			173	0.5%	OOS
			158	0.5%	Concussion and cranial injuries
			160	0.5%	Hearing damage
			120	0.3%	Eye disorders
			76	0.2%	Fatality
			66	0.2%	Skin disease
			43	0.1%	Parasitic disease
			39	0.1%	Heart attack or heart disease
			13	0.04%	Amputation and crushing
			11	0.03%	Cancer
			1	0.00%	Firearm discharge and tinnitus
151	100%		35,255	100%	

Key: OOS: occupational overuse syndrome.

**Table 6:** Injuries by body location.

Mounted Officers			Non-Mounted Officers		
Injuries	Percentage	Location	Injuries	Percentage	Location
21	13.9%	Lower back	4909	13.9%	Knee
11	7.3%	Neck	3525	10.0%	Lower back
11	7.3%	Shoulder	2889	8.2%	Hand
10	6.6%	Ankle	2372	6.7%	Fingers or thumb
10	6.6%	Elbow	2332	6.6%	Shoulder
9	6.0%	Fingers or thumb	1825	5.2%	Lower arm
9	6.0%	Foot	1497	4.3%	Neck
8	5.3%	Hip/s	1390	3.9%	Ankle
7	4.6%	Hand	1326	3.8%	Wrist
7	4.6%	Lower leg	1263	3.6%	Lower leg
6	4.0%	Knee	1248	3.5%	Head
4	2.7%	Upper arm	1171	3.3%	Elbow
4	2.7%	Face	876	2.5%	Upper leg
4	2.7%	Groin	833	2.4%	Face
4	2.7%	Upper leg	810	2.3%	Eye
3	2.0%	Back (multiple)	802	2.3%	Upper arm
3	2.0	Chest	738	2.1%	Foot
3	2.0%	Head	653	1.9%	Other
3	2.0%	Pelvic region	592	1.7%	Chest
3	2.0%	Wrist	549	1.6%	Mouth (not teeth)
2	1.3%	Upper back	498	1.4%	Upper back
2	1.3%	Other	489	1.4%	Back-multiple
2	1.3%	Toe/s	453	1.3%	Hip/s
1	0.7%	Arm lower	329	0.9%	Groin
1	0.7%	Eye	261	0.7%	Ear
1	0.7%	Mouth (not teeth)	257	0.7%	Nose
1	0.7%	Multiple locations	231	0.7%	Abdomen
1	0.7%	Nose	213	0.6%	Internal organs
			212	0.6%	Multiple locations
			199	0.6%	Psychological
			134	0.4%	Toe/s
			106	0.3%	Arm, not classified
			103	0.3%	Nil
			81	0.2%	Leg, not classified
			67	0.2%	Pelvic region
			22	0.1%	Nervous system
151	100%		35,255	100%	

**Table 7:** Injury incident timepoint within a shift.

Mounted Officers			Non-Mounted Officers		
Injuries	Percentage	Hours Worked	Injuries	Percentage	Hours Worked
62	41.1%	<4 h	12,149	34.5%	8–12.5 h
47	31.1%	4–8 h	11,558	32.8%	4–8 h
41	27.2%	8–12.5 h	10,865	30.8%	<4 h
1	0.7%	Unknown	442	1.3%	Unknown
			241	0.7%	12.5 h+
151	100%		35,255	100%	

## Data Availability

Requests for access to the datasets generated and analyzed during this study may be directed to the corresponding author, who will request permission from the stage police force from which the data were provided.
